# Di-μ-bromido-bis­[bromido(di-2-pyridylmethane­diol-κ^2^ *N*,*N*′)copper(II)] dihydrate

**DOI:** 10.1107/S1600536808024203

**Published:** 2008-08-06

**Authors:** Barry L. Westcott, Kristin M. Kopp-Vaughn, Lee M. Daniels, Matthias Zeller

**Affiliations:** a Department of Chemistry and Biochemistry, Central Connecticut State University, New Britain, CT 06050, USA; b Rigaku Americas Corp., 9009 New Trails Dr., The Woodlands, TX 77381, USA; c Department of Chemistry, Youngstown State University, Youngstown, OH 44555, USA

## Abstract

The centrosymmetric title complex, [Cu_2_Br_4_(C_11_H_10_N_2_O_2_)_2_]·2H_2_O, was one of three complexes isolated by slow evaporation of an acetonitrile reaction mixture of CuBr_2_ with di-2-pyridyl ketone (1:1 molar ratio). The title complex contains a 1:1 metal-to-ligand ratio of copper(II) with the hydrated form of the ligand di-2-pyridylmethane­diol. The copper centers are bridged by bromide donors, leading to a Cu—Cu distance of 4.090 (6) Å. The crystals form as non-merohedral twins with two components related by a 180° rotation around the normal to [100]; the selected sample had a twin ratio of 0.63:0.37.

## Related literature

Apart from the title complex, two others were isolated from the reaction mixture and structurally characterized. One complex was reported previously by Parker *et al.* (2000 ▶), the other is reported in the preceding paper by Zeller *et al.* (2008 ▶). For other related structures, see: Wang *et al.* (1986 ▶); Mariezcurrena *et al.* (1999 ▶).
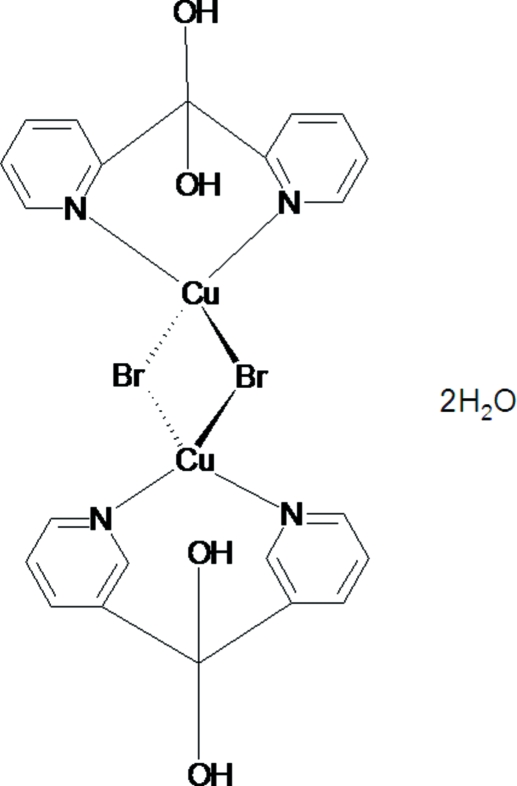


## Experimental

### 

#### Crystal data

[Cu_2_Br_4_(C_11_H_10_N_2_O_2_)_2_]·2H_2_O
*M* *_r_* = 887.18
Monoclinic, 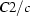

*a* = 21.2685 (7) Å
*b* = 9.1275 (3) Å
*c* = 14.4731 (4) Å
β = 100.749 (2)°
*V* = 2760.34 (15) Å^3^
*Z* = 4
Mo *K*α radiation
μ = 7.38 mm^−1^
*T* = 100 (2) K
0.34 × 0.25 × 0.13 mm

#### Data collection

Rigaku R-AXIS RAPID diffractometer
Absorption correction: multi-scan (*TwinSolve*; Rigaku/MSC, 2002 ▶) *T*_min_ = 0.08, *T*_max_ = 0.38
46376 measured reflections
7508 independent reflections
6192 reflections with *I* > 2σ(*I*)
*R*_int_ = 0.058

#### Refinement

*R*[*F* ^2^ > 2σ(*F* ^2^)] = 0.065
*wR*(*F* ^2^) = 0.198
*S* = 1.13
7508 reflections
185 parameters
2 restraints
H atoms treated by a mixture of independent and constrained refinement
Δρ_max_ = 1.54 e Å^−3^
Δρ_min_ = −1.58 e Å^−3^

### 

Data collection: *CrystalClear* (Rigaku/MSC, 2004 ▶); cell refinement: *TwinSolve* (Rigaku/MSC, 2002 ▶); data reduction: *TwinSolve*; program(s) used to solve structure: *SIR92* (Altomare *et al.*, 1994 ▶); program(s) used to refine structure: *SHELXL97* (Sheldrick, 2008 ▶); molecular graphics: *CrystalStructure* (Rigaku, 2006 ▶); software used to prepare material for publication: *publCIF* (Westrip, 2008 ▶).

## Supplementary Material

Crystal structure: contains datablocks I, global. DOI: 10.1107/S1600536808024203/fj2132sup1.cif

Structure factors: contains datablocks I. DOI: 10.1107/S1600536808024203/fj2132Isup2.hkl

Additional supplementary materials:  crystallographic information; 3D view; checkCIF report

## Figures and Tables

**Table 1:** Hydrogen-bond geometry (Å, °)

*D*—H⋯*A*	*D*—H	H⋯*A*	*D*⋯*A*	*D*—H⋯*A*
O3—H11⋯Br1^i^	0.85 (6)	2.53 (6)	3.361 (5)	166 (9)
O2—H9⋯O1^ii^	0.77 (6)	2.21 (7)	2.961 (7)	147 (9)
O2—H9⋯Br1^ii^	0.77 (6)	2.87 (9)	3.411 (5)	123 (8)
O3—H10⋯Br2	0.85 (6)	2.80 (8)	3.542 (6)	147 (9)

Symmetry codes: (i) 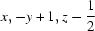; (ii) 
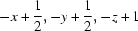
.

**Fig. 1.**
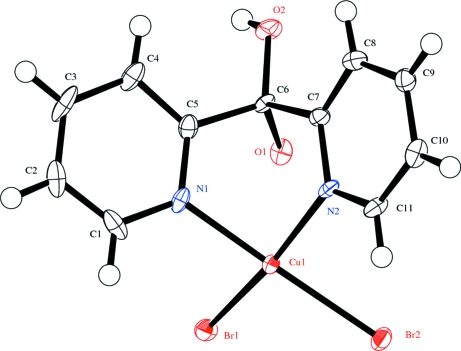
ORTEP view of the unique part of the Cu complex, drawn with 60% probability displacement ellipsoids for the non-H atoms. The water molecule that loosely bridges Br atoms between adjacent complexes is not shown.

**Fig. 2.**
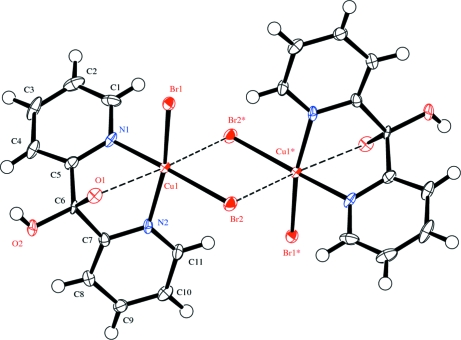
A view of the dimeric unit generated by the association of one of the Br ligands with the Cu atom of an adjacent molecule. The longer interactions of the Br and O atoms are shown as dashed lines. Non-H atoms are drawn with 60% probability ellipsoids.

## supplementary crystallographic information

### Comment

The title compound was one of three Cu–dpkoh complexes isolated from the 1:1 molar mixture of copper(II)bromide and di-2-pyridyl ketone, and was the third isolated from solution. The remaining two strutcures are described in Parker *et al.* (2000) and Zeller *et al.* (2008).

### Experimental

Di-2-pyridyl ketone (dpk) was purchased from Aldrich and used as received. Copper(II) bromide hexahydrate was dried in an oven at 110°C for 48 h before use. DPK (1 mmol) and copper(II) bromide (1 mmol) were combined in 40 ml acetonitrile and stirred for 30 minutes. The resulting olive crystals were isolated after 5 days by slow evaporation of the solution. Prior to harvesting these crystals, crystals of two other distinct complexes were removed by gravity filtration.

### Refinement

The crystals form as non-merohedral twins. All reflections for both domains (44628 total) were integrated with the Rigaku TwinSolve program, to produce 3540 reflections for component 1 only, 3645 for component 2 only, and 815 containing contributions from both components (including systematic absences). The two twin components are related by a 180° rotation around the normal to [1 0 0], given by the matrix (1 0 0.549, 0 - 1 0, 0 0 - 1)

The positions of H atoms bonded to O were allowed to refine with isotropic displacement parameter set equal to the isotropic equivalent value for the attached atom. A mild restraint was applied to the two ligand O—H distances (0.84 Å). Other H atoms were used in calculated positions (C—H 0.95 Å) with isotropic displacement parameter set equal to 1.2 times the the isotropic equivalent value for the attached atom.

### Crystal data

**Table:**

[Cu_2_Br_4_(C_11_H_10_N_2_O_2_)_2_]·2H_2_O	*F*_000_ = 1720
*M**_r_* = 887.18	*D*_x_ = 2.135 Mg m^−^^3^
Monoclinic, *C*2/*c*	Mo *K*α radiation λ = 0.71073 Å
Hall symbol: -C 2yc	Cell parameters from 13358 reflections
*a* = 21.2685 (7) Å	θ = 2.7–29.8º
*b* = 9.1275 (3) Å	µ = 7.38 mm^−^^1^
*c* = 14.4731 (4) Å	*T* = 100 (2) K
β = 100.749 (2)º	Blocks, green
*V* = 2760.34 (15) Å^3^	0.34 × 0.25 × 0.13 mm
*Z* = 4	

### Data collection

**Table:**

Rigaku R-AXIS RAPID diffractometer	7508 independent reflections
Radiation source: fine-focus sealed tube	6192 reflections with *I* > 2σ(*I*)
Monochromator: graphite	*R*_int_ = 0.058
Detector resolution: 10 pixels mm^-1^	θ_max_ = 30.1º
*T* = 100(2) K	θ_min_ = 2.7º
ω scans	*h* = −29→29
Absorption correction: multi-scan(TwinSolve; Rigaku/MSC, 2002)	*k* = −12→12
*T*_min_ = 0.08, *T*_max_ = 0.38	*l* = −20→20
46376 measured reflections	

### Refinement

**Table:**

Refinement on *F*^2^	Secondary atom site location: difference Fourier map
Least-squares matrix: full	Hydrogen site location: difference Fourier map
*R*[*F*^2^ > 2σ(*F*^2^)] = 0.065	H atoms treated by a mixture of independent and constrained refinement
*wR*(*F*^2^) = 0.198	*w* = 1/[σ^2^(*F*_o_^2^) + (0.0753*P*)^2^ + 82.8356*P*] where *P* = (*F*_o_^2^ + 2*F*_c_^2^)/3
*S* = 1.13	(Δ/σ)_max_ = 0.002
7508 reflections	Δρ_max_ = 1.54 e Å^−^^3^
185 parameters	Δρ_min_ = −1.58 e Å^−^^3^
2 restraints	Extinction correction: none
Primary atom site location: structure-invariant direct methods	

### Special details

**Table:**

Geometry. All e.s.d.'s (except the e.s.d. in the dihedral angle between two l.s. planes) are estimated using the full covariance matrix. The cell e.s.d.'s are taken into account individually in the estimation of e.s.d.'s in distances, angles and torsion angles; correlations between e.s.d.'s in cell parameters are only used when they are defined by crystal symmetry. An approximate (isotropic) treatment of cell e.s.d.'s is used for estimating e.s.d.'s involving l.s. planes.
Refinement. Refinement of *F*^2^ against ALL reflections. The weighted *R*-factor *wR* and goodness of fit *S* are based on *F*^2^, conventional *R*-factors *R* are based on *F*, with *F* set to zero for negative *F*^2^. The threshold expression of *F*^2^ > σ(*F*^2^) is used only for calculating *R*-factors(gt) *etc*. and is not relevant to the choice of reflections for refinement. *R*-factors based on *F*^2^ are statistically about twice as large as those based on *F*, and *R*- factors based on ALL data will be even larger.

### Fractional atomic coordinates and isotropic or equivalent isotropic displacement parameters (Å^2^)

**Table:**

	*x*	*y*	*z*	*U*_iso_*/*U*_eq_	
Br1	0.15441 (3)	0.62635 (7)	0.55691 (4)	0.01359 (14)	
Br2	0.02135 (3)	0.54295 (7)	0.38142 (4)	0.01492 (15)	
Cu1	0.08934 (3)	0.41245 (8)	0.50766 (5)	0.00990 (16)	
O1	0.1794 (2)	0.2937 (5)	0.4473 (3)	0.0137 (8)	
H12	0.165 (4)	0.306 (9)	0.394 (3)	0.014*	
O2	0.2056 (2)	0.0447 (5)	0.4596 (4)	0.0163 (9)	
H9	0.241 (2)	0.052 (10)	0.484 (6)	0.016*	
O3	0.1174 (2)	0.3109 (6)	0.2691 (4)	0.0216 (10)	
H10	0.091 (5)	0.391 (10)	0.270 (6)	0.022*	
H11	0.126 (4)	0.334 (10)	0.215 (7)	0.022*	
N1	0.1400 (2)	0.2914 (6)	0.6142 (4)	0.0121 (9)	
N2	0.0544 (2)	0.2175 (6)	0.4509 (4)	0.0107 (9)	
C1	0.1432 (3)	0.3159 (8)	0.7059 (5)	0.0199 (14)	
H1	0.1210	0.3957	0.7238	0.024*	
C2	0.1773 (3)	0.2300 (8)	0.7748 (5)	0.0208 (14)	
H2	0.1792	0.2528	0.8379	0.025*	
C3	0.2088 (3)	0.1091 (9)	0.7493 (5)	0.0231 (15)	
H3	0.2320	0.0486	0.7951	0.028*	
C4	0.2058 (3)	0.0783 (8)	0.6545 (5)	0.0168 (12)	
H4	0.2256	−0.0045	0.6356	0.020*	
C5	0.1724 (3)	0.1751 (7)	0.5888 (4)	0.0134 (11)	
C6	0.1666 (3)	0.1538 (7)	0.4830 (4)	0.0103 (10)	
C7	0.0974 (3)	0.1111 (6)	0.4443 (4)	0.0106 (10)	
C8	0.0795 (3)	−0.0259 (7)	0.4077 (4)	0.0139 (11)	
H5	0.1098	−0.0990	0.4065	0.017*	
C9	0.0145 (3)	−0.0511 (7)	0.3725 (4)	0.0146 (11)	
H6	0.0010	−0.1415	0.3465	0.017*	
C10	−0.0295 (3)	0.0584 (7)	0.3766 (4)	0.0149 (12)	
H7	−0.0727	0.0438	0.3522	0.018*	
C11	−0.0078 (3)	0.1923 (7)	0.4184 (5)	0.0137 (11)	
H8	−0.0374	0.2654	0.4237	0.016*	

### Atomic displacement parameters (Å^2^)

**Table:**

	*U*^11^	*U*^22^	*U*^33^	*U*^12^	*U*^13^	*U*^23^
Br1	0.0089 (2)	0.0146 (3)	0.0168 (3)	0.0002 (2)	0.0011 (2)	−0.0018 (2)
Br2	0.0159 (3)	0.0139 (3)	0.0133 (3)	0.0015 (2)	−0.0015 (2)	0.0027 (2)
Cu1	0.0087 (3)	0.0108 (3)	0.0095 (3)	0.0007 (2)	−0.0003 (2)	−0.0002 (3)
O1	0.012 (2)	0.020 (2)	0.0103 (19)	−0.0023 (17)	0.0046 (16)	0.0041 (17)
O2	0.0081 (19)	0.018 (2)	0.023 (2)	0.0049 (17)	0.0022 (17)	−0.0022 (18)
O3	0.019 (2)	0.033 (3)	0.013 (2)	−0.002 (2)	0.0031 (18)	0.008 (2)
N1	0.007 (2)	0.017 (2)	0.011 (2)	−0.0003 (18)	−0.0015 (17)	0.0040 (19)
N2	0.010 (2)	0.011 (2)	0.011 (2)	0.0045 (18)	0.0031 (17)	0.0051 (18)
C1	0.020 (3)	0.028 (4)	0.013 (3)	−0.007 (3)	0.007 (2)	−0.009 (3)
C2	0.020 (3)	0.032 (4)	0.010 (3)	−0.009 (3)	0.001 (2)	0.001 (3)
C3	0.012 (3)	0.039 (4)	0.018 (3)	0.003 (3)	0.001 (2)	0.018 (3)
C4	0.007 (2)	0.024 (3)	0.019 (3)	0.001 (2)	0.001 (2)	0.009 (3)
C5	0.009 (2)	0.018 (3)	0.012 (3)	−0.001 (2)	0.001 (2)	0.000 (2)
C6	0.003 (2)	0.013 (3)	0.014 (3)	0.0023 (19)	0.0003 (19)	0.001 (2)
C7	0.012 (3)	0.010 (3)	0.010 (2)	0.003 (2)	0.004 (2)	0.003 (2)
C8	0.014 (3)	0.013 (3)	0.016 (3)	0.004 (2)	0.006 (2)	0.002 (2)
C9	0.014 (3)	0.015 (3)	0.014 (3)	−0.001 (2)	0.000 (2)	0.000 (2)
C10	0.010 (3)	0.019 (3)	0.015 (3)	−0.005 (2)	0.000 (2)	0.004 (2)
C11	0.011 (3)	0.012 (3)	0.017 (3)	0.003 (2)	0.000 (2)	0.005 (2)

### Geometric parameters (Å, °)

**Table:**

Br1—Cu1	2.4222 (10)	C2—C3	1.377 (11)
Br2—Cu1	2.4212 (9)	C2—H2	0.9300
Cu1—N1	2.034 (5)	C3—C4	1.390 (10)
Cu1—N2	2.041 (5)	C3—H3	0.9300
Cu1—Br2^i^	3.1138 (10)	C4—C5	1.392 (9)
O1—C6	1.423 (7)	C4—H4	0.9300
O1—H12	0.78 (4)	C5—C6	1.527 (8)
O2—C6	1.378 (7)	C6—C7	1.525 (8)
O2—H9	0.77 (4)	C7—C8	1.382 (9)
O3—H10	0.92 (10)	C8—C9	1.402 (9)
O3—H11	0.86 (10)	C8—H5	0.9300
N1—C1	1.335 (8)	C9—C10	1.377 (9)
N1—C5	1.353 (8)	C9—H6	0.9300
N2—C11	1.339 (8)	C10—C11	1.403 (9)
N2—C7	1.349 (7)	C10—H7	0.9300
C1—C2	1.367 (10)	C11—H8	0.9300
C1—H1	0.9300		
			
N1—Cu1—N2	86.2 (2)	C3—C4—C5	118.0 (7)
N1—Cu1—Br2	175.12 (15)	C3—C4—H4	121.0
N2—Cu1—Br2	90.21 (14)	C5—C4—H4	121.0
N1—Cu1—Br1	91.25 (15)	N1—C5—C4	122.3 (6)
N2—Cu1—Br1	165.08 (15)	N1—C5—C6	115.0 (5)
Br2—Cu1—Br1	93.07 (3)	C4—C5—C6	122.7 (6)
N1—Cu1—Br2^i^	91.44 (15)	O2—C6—O1	113.3 (5)
N2—Cu1—Br2^i^	93.91 (15)	O2—C6—C7	108.0 (5)
Br2—Cu1—Br2^i^	85.54 (3)	O1—C6—C7	109.5 (4)
Br1—Cu1—Br2^i^	100.85 (3)	O2—C6—C5	113.5 (5)
C6—O1—H12	114 (6)	O1—C6—C5	105.3 (5)
C6—O2—H9	114 (7)	C7—C6—C5	107.1 (5)
H10—O3—H11	93 (8)	N2—C7—C8	122.5 (6)
C1—N1—C5	117.9 (6)	N2—C7—C6	114.2 (5)
C1—N1—Cu1	125.7 (5)	C8—C7—C6	123.3 (5)
C5—N1—Cu1	116.5 (4)	C7—C8—C9	118.0 (6)
C11—N2—C7	119.3 (5)	C7—C8—H5	121.0
C11—N2—Cu1	123.6 (4)	C9—C8—H5	121.0
C7—N2—Cu1	117.1 (4)	C10—C9—C8	119.9 (6)
N1—C1—C2	123.4 (7)	C10—C9—H6	120.1
N1—C1—H1	118.3	C8—C9—H6	120.1
C2—C1—H1	118.3	C9—C10—C11	118.6 (6)
C1—C2—C3	119.0 (6)	C9—C10—H7	120.7
C1—C2—H2	120.5	C11—C10—H7	120.7
C3—C2—H2	120.5	N2—C11—C10	121.7 (6)
C2—C3—C4	119.4 (6)	N2—C11—H8	119.2
C2—C3—H3	120.3	C10—C11—H8	119.2
C4—C3—H3	120.3		
			
N2—Cu1—N1—C1	−131.0 (6)	C4—C5—C6—O2	−10.3 (9)
Br1—Cu1—N1—C1	63.9 (5)	N1—C5—C6—O1	49.0 (7)
N2—Cu1—N1—C5	47.9 (5)	C4—C5—C6—O1	−134.4 (6)
Br1—Cu1—N1—C5	−117.3 (4)	N1—C5—C6—C7	−67.6 (7)
N1—Cu1—N2—C11	131.8 (6)	C4—C5—C6—C7	109.0 (7)
Br1—Cu1—N2—C11	−147.0 (5)	C11—N2—C7—C8	−1.5 (9)
Br2—Cu1—N2—C11	−44.9 (5)	Cu1—N2—C7—C8	179.3 (5)
N1—Cu1—N2—C7	−49.1 (5)	C11—N2—C7—C6	179.7 (6)
Br2—Cu1—N2—C7	134.2 (4)	Cu1—N2—C7—C6	0.6 (7)
C5—N1—C1—C2	0.3 (10)	O2—C6—C7—N2	−171.3 (5)
Cu1—N1—C1—C2	179.1 (5)	O1—C6—C7—N2	−47.5 (7)
N1—C1—C2—C3	−2.1 (11)	C5—C6—C7—N2	66.6 (7)
C1—C2—C3—C4	0.8 (11)	O2—C6—C7—C8	10.0 (8)
C2—C3—C4—C5	2.2 (10)	O1—C6—C7—C8	133.8 (6)
C1—N1—C5—C4	2.9 (9)	C5—C6—C7—C8	−112.1 (7)
Cu1—N1—C5—C4	−176.0 (5)	N2—C7—C8—C9	3.0 (9)
C1—N1—C5—C6	179.6 (6)	C6—C7—C8—C9	−178.4 (6)
Cu1—N1—C5—C6	0.6 (7)	C7—C8—C9—C10	−2.0 (10)
C3—C4—C5—N1	−4.2 (10)	C7—N2—C11—C10	−1.0 (10)
C3—C4—C5—C6	179.4 (6)	C9—C10—C11—N2	1.9 (10)
N1—C5—C6—O2	173.1 (5)		

Symmetry codes: (i) −*x*, −*y*+1, −*z*+1.

### Hydrogen-bond geometry (Å, °)

**Table:**

*D*—H···*A*	*D*—H	H···*A*	*D*···*A*	*D*—H···*A*
O3—H11···Br1^ii^	0.85 (6)	2.53 (6)	3.361 (5)	166 (9)
O2—H9···O1^iii^	0.77 (6)	2.21 (7)	2.961 (7)	147 (9)
O2—H9···Br1^iii^	0.77 (6)	2.87 (9)	3.411 (5)	123 (8)
O3—H10···Br2	0.85 (6)	2.80 (8)	3.542 (6)	147 (9)

Symmetry codes: (ii) *x*, −*y*+1, *z*−1/2; (iii) −*x*+1/2, −*y*+1/2, −*z*+1.
